# When Wellness Stress Detection Enters Healthcare Workflows: An Iso-Heart-Rate, Cross-Corpus Evaluation of Wrist Wearables

**DOI:** 10.3390/healthcare14152434

**Published:** 2026-08-06

**Authors:** Nowon Kwon, Seonkyung Yoon, Jungyi Hur, Hye Seung Kang

**Affiliations:** 1 AI-Driven Medical Innovation Center, Kyungpook National University Hospital, Daegu 41944, Republic of Korea; nowonkwon25@gmail.com; 2 Department of Nursing, Saekyung University, Yeongwol 26239, Republic of Koreahjy52@saekyung.ac.kr (J.H.)

**Keywords:** digital health, healthcare, wearable sensors, nursing workforce, electrodermal activity, stress detection, heart-rate confounding, clinical informatics, domain adaptation, alert fatigue

## Abstract

**Background/Objectives:** Benchmark performance does not establish that a wrist-wearable stress measure is valid in healthcare workflows. We tested whether binary stress-versus-non-stress performance reflects stress-specific physiology rather than heart-rate (HR) arousal, as well as whether models generalize from wellness-like corpora to a clinical workforce context. **Methods:** We analyzed three public wrist Empatica E4 corpora: Wearable Stress and Affect Detection (WESAD), Stress-Predict, and a real-world Nurse dataset. An iso-heart-rate (iso-HR) evaluation balanced the HR within subjects before measuring non-cardiac discriminability. We compared a continuous skin conductance response (SCR) count with logistic regression, gradient boosting, four unsupervised domain-adaptation methods, and an accelerometer context-aware variant, under leave-one-subject-out and leave-one-dataset-out evaluation with subject-cluster bootstrap intervals. **Results:** The single SCR score was statistically indistinguishable from logistic regression in paired cross-corpus tests, and no adaptation method moved a weak corpus into a usable range: across all evaluated models, the transfer area under the receiver operating characteristic curve (AUROC) was approximately 0.90 with WESAD as the target but 0.52–0.57 with Stress-Predict or Nurse. Adding activity context raised the within-corpus AUROC yet lowered cross-corpus transfer, because the movement–stress association reverses across protocols. On Nurse, source-trained models reached AUROC 0.541–0.559 against prevalence 0.796 with poor calibration, and conditioning on movement or on distance to the source distribution removed the residual discrimination. Iso-HR evidence was diagnostic mainly in WESAD. **Conclusions:** Neither shallow unsupervised adaptation nor wrist activity context established reliable transfer to Nurse, and sparse retrospective labels prevent attributing this solely to physiological non-transfer. Cross-corpus and iso-HR controls, not model complexity, determine what can be concluded for healthcare workflows.

## 1. Introduction

Wearable biosensors and affective computing have made continuous physiological stress monitoring increasingly visible in digital health, public health, and occupational wellness. Wrist-worn devices such as the Empatica E4 and consumer smartwatches can collect autonomic signals unobtrusively during daily life, including heart rate (HR), heart-rate variability (HRV), electrodermal activity (EDA), and skin temperature [1,2]. Motivated by this technological promise, machine-learning studies have optimized stress classification pipelines on laboratory datasets. For example, WESAD reported 93.12% accuracy for subject-independent chest-sensor classification under leave-one-subject-out validation [3]. Other controlled and real-world studies reported lower or context-dependent performance, showing that activity and setting remain important even when within-study results appear promising [4,5]. These results have helped establish wearable stress detection as a plausible digital health application, and related Healthcare studies have examined wearable stress management and context-aware smart-band feedback in real-life settings [6,7].

Yet, a digital health signal becomes clinically meaningful only after its context of use has been specified and its measurement validity has been established. Digital medicine frameworks distinguish technical verification, analytical validation, and clinical validation, and they emphasize that biometric monitoring technologies should be fit for the intended population and setting before their outputs are interpreted as health measures [8]. Similarly, patient- and user-centered digital-measure frameworks caution against proliferating low-value measures that are technically measurable but burdensome or weakly connected to decisions that matter in care [9]. For stress monitoring, this distinction is central: a wearable score that is acceptable as a private wellness cue may be inappropriate as an input to clinical workflow, staffing, or occupational-health decisions.

For healthcare context-of-use validation, however, the relevant question is not only whether a model can classify stress within a familiar benchmark. The harder question is whether a model trained under wellness or laboratory conditions remains valid when it is moved into an active clinical workforce. These concerns are especially salient in safety-critical and emotionally intensive occupations, where stress monitoring is not merely a private wellness function but may become linked to work design, occupational health, staffing, care quality, or patient safety. Frontline healthcare work is a clear example. The coronavirus disease 2019 (COVID-19) context made psychological strain among healthcare workers highly visible [10,11], and nursing stress and burnout drew particular attention because their implications extend beyond individual well-being to workload, workforce retention, care quality, and patient safety [12,13]. At the same time, clinical work is physiologically complex: cognitive–emotional strain occurs alongside walking, patient handling, and shift-dependent physical demands [14], as well as alarms, interruptions, and urgent bedside tasks [15,16]. In a descriptive seven-shift case study, HR monitoring classified 87% of nurses’ time as active, whereas accelerometry classified 27%; the authors also note that stress can elevate HR during tasks involving little or no movement [17]. Nursing is therefore a useful boundary case for wrist-wearable stress detection, not because this study seeks a nurse-specific predictor, but because it provides a demanding context-of-use test in which a wellness stress score may lose validity.

This translational risk is amplified by a physiological confound. Stress tasks often elevate HR, but HR is not specific to psychological stress. Physical activity, posture, thermoregulation, and routine clinical work can also increase HR and alter wrist physiology [18,19]. A wearable classifier may therefore achieve high apparent performance by separating high-arousal movement from low-arousal rest, rather than detecting stress-specific physiology. In a hospital ward, this distinction matters: a model that mistakes patient lifting or urgent movement for psychological strain may generate operational noise rather than protective insight. Thus, before consumer smartwatch stress scores or wellness stress dashboards are scaled into healthcare workforce monitoring or organizational wellness programs, their physiological boundary conditions need to be tested explicitly under the intended context of use [8,17], while organizational adoption also requires attention to implementation barriers, governance, and acceptability [20].

A second barrier is cross-corpus generalizability. Even leave-one-subject-out validation within one dataset evaluates new people under the same stressor type, device configuration, labeling scheme, and preprocessing assumptions. It does not establish that models trained under one laboratory implementation of social-evaluative or cognitive challenge will transfer to naturalistic hospital work. This evolution is also reflected in the public datasets available for wrist-based stress research. Controlled corpora such as Wearable Stress and Affect Detection (WESAD) support benchmark development, whereas more naturalistic datasets test whether stress models survive changes in stressor type, activity context, and labeling conditions. Recent work has emphasized that stressor type can dominate cross-dataset generalizability in physiological stress detection [21]. Systematic reviews likewise identify limited statistical power, inconsistent labeling, and weak evidence for generalization as recurring problems in wearable stress research [22]. Recent studies have begun to address this gap using real-life ecological momentary assessment (EMA) designs, office-like work simulations, cross-dataset HRV analyses, and cross-context/domain-adaptation frameworks [23,24,25,26]. New datasets that explicitly combine acute stress and exercise further show why physical arousal must be handled as a competing context rather than a nuisance variable [27]. From a Healthcare perspective, the Nurse corpus should therefore not be treated simply as a difficult or low-performing dataset. It is an ecologically demanding test of whether models trained or validated in wellness-like conditions retain meaning in clinical workforce settings.

In this study, we use three public wrist Empatica E4 corpora to define these boundaries: Wearable Stress and Affect Detection (WESAD), Stress-Predict, and a real-world Nurse dataset collected from hospital nurses [3,28,29]. We do not propose a new deployable stress monitor. Instead, we ask three translational questions. (RQ1) Does multivariate machine learning provide reliable value beyond a single interpretable skin conductance response (SCR) feature? (RQ2) Does stress-detection performance transfer across stressor types and into the Nurse corpus? (RQ3) Does HR-level confounding explain the observed corpus differences, and in which corpora is an iso-HR decomposition diagnostic? To answer these questions, we introduce an iso-heart-rate (iso-HR) evaluation that balances HR within subject before measuring the residual discriminability of non-cardiac signals. The purpose is to turn laboratory-to-clinic generalization into a measurable validity problem: before wearable stress outputs are treated as actionable in healthcare workflows, they should survive HR control and cross-corpus testing.

## 2. Materials and Methods

### 2.1. Analysis Overview

**Figure 1:**
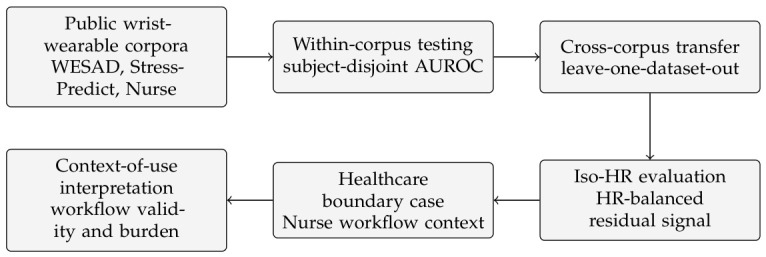
Context-of-use validation workflow for wearable stress classification. The workflow separates benchmark performance from healthcare translation by adding cross-corpus transfer, HR control, and workflow-sensitive interpretation before considering clinical workforce use.

The analysis was designed to make the translation problem visible rather than to maximize a single benchmark score. Figure 1 shows the context-of-use validation workflow, and Table 1 summarizes the full pipeline from public datasets to clinically oriented interpretation.

### 2.2. Datasets

We use three publicly available wrist Empatica E4 corpora (Table 2). WESAD [3] provides laboratory social-evaluative stress (TSST [30]) versus baseline/meditation. Stress-Predict [28] provides a Stroop color-word task and a less confrontational implementation of the TSST interview versus relaxation. Following the published time logs and the analysis code, positive windows comprise Stroop and interview intervals, negative windows comprise baseline and relaxation, and the Hyperventilation Provocation Test and questionnaire intervals are excluded; labels are constructed from the protocol time logs rather than from a precomputed label column. Nurse [29] provides real-world hospital recordings with retrospective self-reported stress levels (we contrast the survey extremes, high-stress vs. no-stress, excluding the ambiguous middle level). From each corpus, we form a binary stress (+)/non-stress (−) task using the wrist E4 streams (blood-volume pulse 64 Hz, EDA 4 Hz, skin temperature 4 Hz, accelerometer 32 Hz); device characteristics are as validated previously [31].

### 2.3. Operational Definition of Healthcare Context-of-Use

For this analysis, healthcare context-of-use is defined as the condition in which a wearable stress output would be interpreted within an active clinical workforce workflow rather than as a private wellness reflection. This definition has three components. First, the signal must retain meaning under the physical conditions of care work, including walking, standing, lifting, urgent movement, thermal variation, and device-fit changes during long shifts. Second, the signal must remain interpretable under cognitive and emotional conditions that differ from laboratory stressors, such as alarms, interruptions, multitasking, time pressure, patient deterioration, and interpersonal conflict. Third, the output must have a plausible action pathway: it may enter a dashboard, alert, staffing conversation, occupational-health review, or well-being intervention. These requirements make healthcare context-of-use stricter than ordinary within-dataset classification, because errors do not merely reduce an AUROC score; they can add workflow noise, staff concern, or misleading operational interpretation.

The three corpora are therefore not treated as interchangeable samples from one stress population. Instead, they operationalize three increasingly demanding context-of-use conditions for a wrist-based stress measure. WESAD represents benchmark validity: whether wrist physiology can detect a strong, protocol-defined social-evaluative stressor. Stress-Predict represents stressor-shift validity: whether a model or feature remains useful when the stressor protocol changes to a Stroop task and a less confrontational TSST implementation. The Nurse corpus represents healthcare workflow validity: whether a wellness-like stress output retains meaning when physiological arousal is embedded in real clinical work and retrospective self-report. Under this operational definition, the Nurse corpus is not included because the study aims to build a nurse-specific predictor. It is included because it is a boundary case for healthcare translation. Table 3 states this role explicitly.

### 2.4. Windowing and Features

Signals are segmented into 64 s windows with 50% overlap. EDA is decomposed into tonic and phasic components with cvxEDA [32], as implemented in NeuroKit2 [33]; skin-conductance responses (SCRs) are counted as local maxima of the phasic signal [34]. From each window, we compute four feature families: (i) HR level (mean HR, mean inter-beat interval (IBI)); (ii) cardiac dynamics/variability: HR slope, range, and curvature; HRV indices (root mean square of successive differences (RMSSD), standard deviation of normal-to-normal intervals (SDNN); and percentage of successive heartbeat intervals differing by >50 ms (pNN50), with a Malik-rule artifact flag) [35]; (iii) non-cardiac: tonic and phasic EDA (SCR count, amplitude, area, decay) and skin temperature (mean, slope); and (iv) activity/movement, derived from the 32 Hz three-axis wrist accelerometer as the vector-magnitude mean, standard deviation, Euclidean norm minus one, and mean absolute jerk. All three corpora record wrist accelerometry on the same Empatica scale, so the activity block is computed identically in each. Families (i)–(iii) enter the classification models; family (iv) is used for the movement stratification (Section 3.6) and for the context-aware model described below and never enters the primary feature set. The single most discriminative EDA feature in within-fold permutation importance is the SCR count, which we use as the single-feature predictor. It is therefore the best single EDA feature selected within training folds rather than an unexamined a priori feature choice. Its decision direction is fixed a priori (more SCRs → more stress); it is never flipped using test labels, so a below-chance area under the receiver operating characteristic curve (AUROC) is reported as such. SCR count remains a continuous score for the primary AUROC analysis, so no classification threshold is selected. For robustness, we repeat the full protocol with non-overlapping 64 s windows (64 s step) and with 300 s windows at 50% overlap (150 s step). At both window lengths, we also compare otherwise identical all-feature models with models omitting six HRV/quality variables: RMSSD, SDNN, pNN50, valid-IBI count, IBI coefficient of variation, and artifact fraction. For cross-corpus transfer, we additionally restrict to a transfer-robust set of eight device- and ambient-invariant quantities: phasic SCR count, mean and maximum SCR amplitude, phasic area, tonic (skin-conductance-level) slope, EDA decay, temperature slope, and HR slope. Absolute means (mean HR, mean skin conductance, mean temperature) are excluded because they do not transfer across devices. The context-aware variant evaluated in Section 3.4 appends the four activity features to these eight, giving twelve inputs.

### 2.5. Iso-HR Matching and the HR-Leakage Index

To separate stress-specific physiology from non-specific arousal, we balance HR before evaluation. Within each subject, windows are binned into 5 bpm HR bins, and the majority class is randomly subsampled so that the two classes have identical HR distributions per bin. This iso-HR matched set is a study-design subsampling that uses HR and labels only to balance counts and *never* participates in model fitting. Because the subsample is random, the matching is repeated over multiple seeds, and we report the stability of the resulting estimates (Section 3.6).

Operationally, matching is performed on whole precomputed windows, separately for each subject:

1. Assign every intact window to a 5 bpm bin using its mean HR;
2. Within each subject–bin combination, set the retained count to the smaller of the stress and non-stress counts;
3. Sample that number of complete windows from each class without removing or splicing any signal samples;
4. Concatenate the retained windows and perform subject-wise evaluation.

Bins containing only one class contribute no windows. Features, including HRV, are not recomputed after matching because the unit of selection is the intact window. This procedure can remove many windows when within-subject class overlap in HR is limited; we therefore report matched sample sizes and seven-seed stability.

On the matched set, we evaluate three nested feature sets: all features (including HR level); HR-level-free (all features except mean HR and mean IBI; this still contains cardiac dynamics and HRV, so it is not “non-cardiac”); and non-cardiac (EDA and temperature only). We define the HR-leakage index as the matched-set AUROC of the all-feature model minus that of the HR-level-free model; a value near zero means that HR level contributes little once the other signals are present. We emphasize that a near-zero index is informative only where a discriminative signal exists at all; in a corpus where every feature set is near chance, the index is near zero trivially (a floor effect) and carries no evidence about confounding.

### 2.6. Models and Evaluation

We compare two groups of predictors, plus two further variants defined below. The reference group contains (i) a single feature, the SCR count used directly as a score with no training, which makes it corpus-invariant by construction: its within-corpus and cross-corpus AUROC on a given target are identical because nothing is fit; (ii) logistic regression (linear; per-fold median imputation and standardization fit on training data only); and (iii) histogram gradient boosting (non-linear, with native missing-value handling).

The unsupervised adaptation group contains four methods that see the target’s feature distribution but never its labels: CORAL [36], which aligns second-order statistics of the source to those of the target; subspace alignment [37], which maps the source principal axes onto the target principal axes; transfer component analysis [38], which learns a kernel subspace minimizing the maximum mean discrepancy between domains; and importance-weighted logistic regression, which reweights source windows by a domain-classifier density ratio to correct covariate shift [39,40,41]. All four are transductive: they use the unlabeled target feature distribution, which the reference models never see, so their failure to beat those models is a conservative result. Transfer component analysis requires kernel and dimensionality choices without a target-label-free selection rule. Subspace alignment has a theoretically motivated dimensionality rule [37], but it was derived for high-dimensional visual descriptors and is uninformative in our eight-dimensional feature space. We therefore sweep both methods and report the setting that maximizes target AUROC: 2–8 components for subspace alignment, and the same component range crossed with three radial basis function (RBF) bandwidths (0.5×, 1× and 2× the median-distance heuristic) for transfer component analysis, because both conventions appear in the literature and the result is sensitive to the choice. Each reported value is therefore an optimistic upper bound that no honest selection rule can exceed, so a conclusion that survives it does not depend on hyperparameter selection. Transfer component analysis is fitted on a fixed-seed subsample of 1000  windows per domain because a full kernel eigendecomposition over all windows is not tractable; the learned map is then applied to every window.

The context-aware variant (Section 3.4) appends the four wrist-accelerometer activity features to the eight transfer features and is otherwise identical to logistic regression and gradient boosting. To separate adaptation limits from target-signal limits, we additionally report a supervised reference in which k∈{2,4,8} target subjects are labeled and added to the training set, with evaluation on the remaining target subjects over 20 fixed-seed draws; this is not leave-one-dataset-out and is reported separately as an upper bound. Within-corpus performance uses leave-one-subject-out (LOSO) cross-validation; cross-corpus generalization uses leave-one-dataset-out (LODO; train on the union of the other corpora, test on the held-out corpus). The area under the receiver operating characteristic curve (AUROC) is the primary metric. For leave-one-dataset-out target estimates and paired model differences, uncertainty is estimated by a subject-level cluster bootstrap (1000 resamples) [42]; the within-corpus LOSO table reports point estimates. Significance against chance is assessed by a cluster-aware permutation test that retains the fixed target scores and subject set, shuffles labels within each subject, and recomputes the statistic. This preserves subject structure and class balance; *p*-values across the family of cross-corpus tests are Holm-corrected. The two procedures answer different questions: the bootstrap quantifies generalization to new subjects, whereas the within-subject permutation, which holds the subject set fixed, tests only whether a within-corpus association exceeds chance. We therefore treat the subject-cluster bootstrap as the primary, more conservative summary for generalization claims and use the permutation as a secondary check. To compare models (rather than against chance), we use a paired subject-cluster bootstrap of the AUROC difference and call two models statistically indistinguishable when the 95% interval of their difference contains zero.

For the imbalanced Nurse target, we additionally report average precision (AP), Brier score, 10-bin expected calibration error (ECE), balanced accuracy, sensitivity, and false-positive rate (FPR). Logistic-regression and boosting operating metrics use a fixed probability threshold of 0.5 chosen before inspecting Nurse labels; it is not tuned on the target. To describe, rather than causally explain, domain discrepancy, each transfer feature is compared between corpus pairs using one-dimensional Wasserstein distance divided by the pairwise pooled interquartile range. Stress-Predict label alignment is assessed by shifting all protocol intervals by −64, −32, 0, +32, and +64 s while keeping every other analysis choice fixed. The absolute movement gate of 0.0796 g is an externally derived distributional threshold, not a literature reference value: it is the 95th percentile of accelerometer-magnitude SD across 2851 64-second stress windows in the independent PhysioNet induced-stress and structured-exercise dataset [43], and it is applied unchanged without tuning on WESAD, Stress-Predict, or Nurse.

### 2.7. Reproducibility and Leakage Control

The only train/test partition is by subject; subjects never appear on both sides (enforced by an assertion, with split sessions merged so one subject cannot enter both folds). Iso-HR matching never fits a model, and gradient boosting handles missing values natively, so no scaler or imputer statistics leak from training to test. We ran an adversarial code review for cross-subject leakage and found none; a within-subject look-ahead remains because EDA decomposition is computed once per session, but this is not a cross-subject leak and does not inflate LOSO estimates. As a negative control, we permuted labels within each subject, preserving every subject’s class balance, and re-ran the whole within-corpus pipeline unchanged over five permutations. It returns AUROC 0.482±0.023 on WESAD and 0.481±0.019 on Stress-Predict, so no label information reaches the model through feature construction, the split, or the evaluation path. On Nurse the same control returns 0.298±0.011. A below-chance value cannot indicate leakage, which would inflate rather than deflate the statistic; it reflects the fact that pooled Nurse AUROC is governed by between-subject score offsets against strongly unequal per-subject prevalences—the same structure that Section 3.8 isolates by conditioning. Consequently, the empirical null for this pooled Nurse statistic is near 0.30, not 0.50, under the within-subject permutation scheme. Pooled Nurse values of 0.541–0.559 therefore should not be interpreted as marginally above chance merely because they exceed 0.50; the subject-cluster bootstrap and the stratified analyses in Section 3.8 carry the interpretation. All bootstrap resampling is by subject and all permutations remain within subject, so overlapping windows never cross train/test or resampling blocks. Analyses used Python 3.11.13 with scikit-learn 1.9.0, NumPy 2.4.6, SciPy 1.17.1, pandas 2.3.3, NeuroKit2 0.2.13 and CVXOPT 1.3.3; the complete environment, with exact pins for every dependency, is specified in env.yaml. All supported commands run through versioned shell entry points in scripts/, and all randomness uses fixed, reported seeds. Analysis code, the exact environment, and versioned entry points are openly available in the public repository and Zenodo archive identified in the Data Availability Statement.

## 3. Results

### 3.1. A Single Skin-Conductance Feature Is on Par with Machine Learning

Within each corpus (Table 4; feature families and the transfer-robust set are defined in Section 2.4), a single SCR feature is on par with linear and non-linear machine learning where the signal is strong: on WESAD, it reaches AUROC 0.897 versus 0.876 (logistic) and 0.886 (boosting). Machine learning shows a modest edge only on Stress-Predict, 0.628/0.633 vs. 0.563, Δ≈0.07), where all models nonetheless remain weak (<0.65); on the real-world corpus (Nurse) every model is near chance and the single feature (0.553) is not beaten by logistic regression (0.450, i.e., below chance under LOSO, indicating subject-level heterogeneity rather than learnable structure). Thus, added model capacity yields no consistent advantage over the single interpretable feature; where it appears to help, the absolute performance is still too low to be clinically useful. This points to a univariate physiological effect rather than a machine-learning achievement.

**Table 5:** Leave-one-dataset-out AUROC (train on the other corpora, test on target). The 95% subject-bootstrap confidence intervals shown for the single/logistic models. WESAD is clearly above chance, Stress-Predict is marginally above 0.5, and the Nurse interval includes 0.5.

Test Corpus	Single (SCR)	Logistic	Boosting	CORAL
WESAD	0.897 [0.832–0.953]	0.890 [0.814–0.949]	0.737	0.879
Stress-Predict	0.563 [0.525–0.607]	0.564 [0.530–0.602]	0.558	0.564
Nurse	0.553 [0.407–0.694]	0.541 [0.423–0.656]	0.559	0.542

### 3.2. Cross-Corpus Transfer Is Poor for Every Model Evaluated

**Figure 2:**
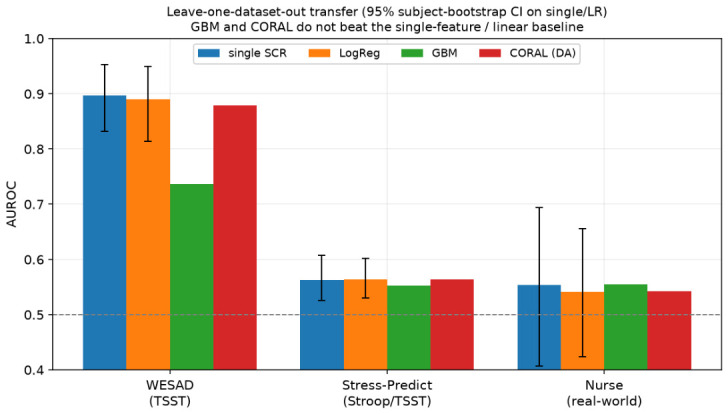
Leave -one-dataset-out AUROC by model and target corpus (error bars: 95% subject-bootstrap CI for single and logistic models). Under the evaluated models, gradient boosting and CORAL do not exceed the single-feature/linear baseline. Figure legend abbreviations: LogReg, logistic regression; GBM, gradient boosting; DA, domain adaptation.

Leave-one-dataset-out transfer (Table 5, Figure 2) is strong only when the target corpus is WESAD and weak otherwise. The single feature is corpus-invariant by construction, so its 0.897 on WESAD is identical to its within-corpus value; notably, the logistic model trained on the other corpora also transfers to WESAD almost as well (0.890), because WESAD is the corpus that carries a strong, well-aligned signal in the first place, not because cross-corpus training adds information. Non-linear boosting is no better than, and often worse than, linear logistic regression (AUROC 0.737 vs. 0.890 with WESAD as target), and the unsupervised domain-adaptation method CORAL recovers the boosting loss but does not exceed the simple linear or single-feature baseline in the evaluated comparisons. Section 3.3 extends this comparison to three further unsupervised adaptation methods and Section 3.4 to a wrist-accelerometer context-aware model; neither recovers transfer. What remains untested is adversarial representation learning [44] and richer non-wearable context such as shift metadata, workload, or alarm exposure. Testing model equivalence directly rather than relying on overlapping intervals, paired subject-bootstrap differences show the single feature to be statistically indistinguishable from logistic regression in every corpus (e.g., WESAD single − logistic =+0.007, 95% confidence interval (CI) [−0.003,+0.022]), and from CORAL; likewise, against gradient boosting, it is indistinguishable on the two weak corpora but significantly better on WESAD (+0.165, [+0.057,+0.278]).

Subject-bootstrap 95% confidence intervals place WESAD clearly above chance, Stress-Predict only marginally above 0.5, and Nurse across 0.5. The Nurse interval is especially wide because the target contains only 15 subjects, limiting subject-level power. The cluster-aware permutation test (within-subject label shuffle, Holm-corrected) confirms WESAD transfer for both the single and logistic models (p=0.003). It also reaches significance for the weak corpora, but there it reflects the large number of windows rather than reliability across people. For Nurse in particular, the structured empirical null is near 0.30, so neither exceeding 0.50 nor the permutation result establishes reliable discrimination. The subject-cluster bootstrap, which resamples people, and the within-context stratification in Section 3.8 are the appropriate summaries for interpretation. By those standards, WESAD is clearly above chance, Stress-Predict is marginal, and Nurse is not distinguishable from chance.

### 3.3. Four Unsupervised Adaptation Methods Do Not Recover Transfer

**Figure 3:**
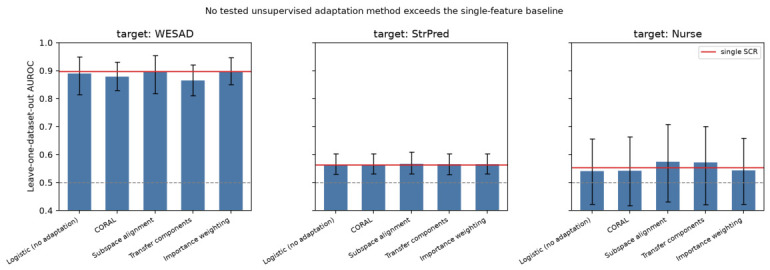
Leave -one-dataset-out AUROC for each unsupervised adaptation method and target corpus (error bars: 95% subject-bootstrap CI). The red line is the single-SCR reference, and the dashed line is chance. Adaptation does not move Stress-Predict or Nurse into a usable range. StrPred denotes Stress-Predict in the figure.

Because CORAL alone cannot represent unsupervised domain adaptation, we repeated the leave-one-dataset-out protocol with three further methods that also see the unlabeled target distribution (Table 6, Figure 3). For subspace alignment and transfer component analysis we report the component count that maximizes target AUROC, an optimistic upper bound; the full grids are given in the deposited result ledger.

No method moved a weak corpus into a usable range. With Stress-Predict as target, all six predictors fall between 0.563 and 0.567, and every paired difference against the single feature contains zero. With WESAD as target, no adaptation method meaningfully exceeded the single feature (the largest advantage was under 0.001 AUROC), and none was significantly worse; the weakest, transfer component analysis, reached 0.865 against 0.897 with a paired difference of +0.030, 95% CI [−0.000,+0.065].

Nurse is the only target where an adaptation method significantly exceeded the single feature, that is, where the paired difference excluded zero: best-case subspace alignment reached 0.574 against 0.553, and the paired difference was −0.020, 95% CI [−0.039,−0.002]. We report this explicitly, but it does not change the operational conclusion. The gain is obtained by selecting the component count on target performance; across the untuned grid the same method spans 0.538–0.574. Its subject-bootstrap interval, [0.431,0.707], still includes chance, so transfer to Nurse remains undemonstrated at the level of new subjects.

Because an unsupervised method can only exploit structure the target actually contains, we also asked what happens when target labels are supplied outright. We trained on both source corpora plus *k* labeled target subjects and evaluated on the remaining subjects over 20 fixed-seed draws. Within each draw, we re-scored the source-only model on the same held-out subjects, so the reported gain isolates the effect of supplying labels rather than a change of evaluation cohort. With eight labeled subjects, the mean paired gain is +0.002 for WESAD, +0.001 for Stress-Predict and +0.029±0.024 for Nurse. Labeling more than half of the available Nurse participants therefore buys a small and unstable improvement that leaves the corpus near chance, which places the limitation in the target signal and labels rather than in the choice of adaptation method.

### 3.4. Activity Context Helps Within a Corpus and Hurts Across Corpora

**Figure 4:**
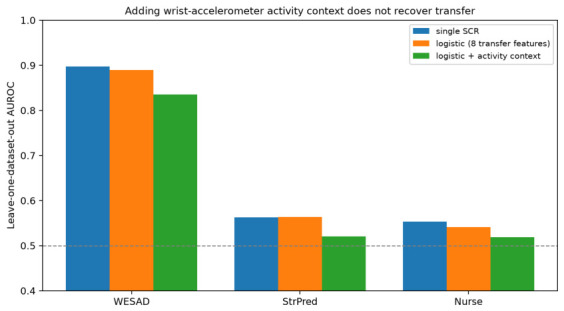
Leave -one-dataset-out transfer with and without wrist-accelerometer activity context. Adding activity features lowers transfer AUROC for every target corpus, although the same features raise within-corpus performance. The dashed horizontal line indicates chance-level AUROC of 0.5.

The wrist accelerometer is the one context channel available in all three corpora, so we tested the context-aware model the translation argument implies: the eight transfer features plus the four activity features (Table 7, Figure 4).

Within a corpus, activity context raises every estimate. Leave-one-subject-out AUROC rises from 0.876 to 0.898 (logistic) and 0.886 to 0.930 (boosting) on WESAD, from 0.628 to 0.681 and 0.633 to 0.671 on Stress-Predict, and from 0.450 to 0.468 (logistic) and 0.489 to 0.572 (boosting) on Nurse. The Nurse logistic model nonetheless stays below chance in both variants, so the improvement there is relative, not useful.

Across corpora, the same features hurt. Leave-one-dataset-out logistic AUROC falls from 0.890 to 0.836 on WESAD, 0.564 to 0.520 on Stress-Predict, and 0.541 to 0.519 on Nurse. Boosting falls on the two weak corpora as well (0.558 to 0.512 and 0.559 to 0.545); its WESAD value rises from 0.737 to 0.762 but remains far below both the single feature and plain logistic regression. The reason is visible in the association between movement and the stress label, which does not share a direction across corpora: mean absolute jerk alone separates stress from non-stress at AUROC 0.828 in WESAD, where the speaking-and-standing protocol makes stress the more active condition, but at 0.481 in Stress-Predict, whose retained Stroop and interview tasks are largely seated, and 0.590 in Nurse. A model trained on activity alone transfers to WESAD at 0.792, confirming that most of what activity contributes is protocol identity rather than stress physiology.

Activity context is therefore not a free improvement. It raises within-corpus performance by encoding what each protocol asked participants to do, and that is precisely the information that does not transfer to a new setting.

### 3.5. Robustness to Windowing, HRV, and Label Alignment

Removing window overlap leaves the leave-one-dataset-out pattern essentially unchanged (Table 8). With 300 s windows, WESAD remains the only strong target, while Stress-Predict and Nurse remain weak. The corresponding subject-bootstrap intervals are WESAD 0.859–0.974, Stress-Predict 0.520–0.611, and Nurse 0.383–0.742 for the single score; the last interval remains especially wide because only 15 Nurse subjects are available.

The direct HRV ablation reaches the same conclusion (Table 9). Including RMSSD, SDNN, pNN50, valid-IBI count, IBI coefficient of variation, and artifact fraction changes the LOSO AUROC by at most 0.017 at 64 s and 0.010 at 300 s. Longer windows therefore do not reveal a previously hidden HRV contribution that rescues the weak corpora. However, 300 s windows reduce the available windows from 1133/2846/9226 to 166/557/1770, so the long-window estimates are sensitivity checks rather than replacements for the primary analysis.

**Figure 5:**
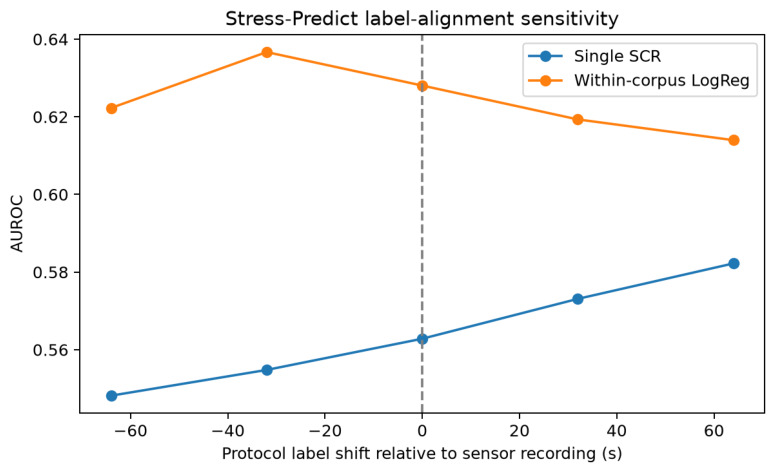
Stress-Predict label-alignment sensitivity. All protocol intervals are shifted by the amount on the horizontal axis, while every other analysis choice is held fixed; the dashed line marks the primary alignment. No shift within one window length produces strong discrimination.

Shifting every Stress-Predict protocol interval by up to one primary window in either direction does not change the qualitative result. Across shifts from −64 to +64 s, single-SCR AUROC ranges from 0.548 to 0.582 and within-corpus logistic AUROC from 0.614 to 0.637 (primary alignment: 0.563 and 0.628). The two series move in opposite directions (Figure 5): the single-SCR score increases monotonically as the protocol labels are moved later, whereas the within-corpus logistic model peaks at −32 s. Alignment uncertainty can therefore contribute to the point estimate but does not explain the weak discrimination by itself.

**Figure 6:**
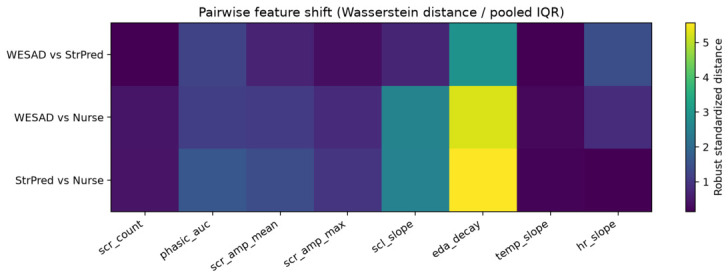
Pairwise distribution shift for the eight transfer features. Each cell is a one-dimensional Wasserstein distance divided by the pairwise pooled interquartile range (IQR); larger values indicate greater marginal discrepancy, not a causal contribution to error. StrPred denotes Stress-Predict in the figure.

Pairwise robust-standardized Wasserstein distances show measurable feature shift (Figure 6). Mean distance is 0.96 for WESAD–Stress-Predict, 1.55 for WESAD–Nurse, and 1.61 for Stress-Predict–Nurse; the largest individual shifts occur in EDA decay, skin conductance level (SCL) slope, and phasic area. These descriptive discrepancies identify candidate measurement/context differences but cannot separate stressor type, device fit, population, and labeling quality causally.

### 3.6. Iso-HR Evidence Is Diagnostic Mainly in WESAD

**Figure 7:**
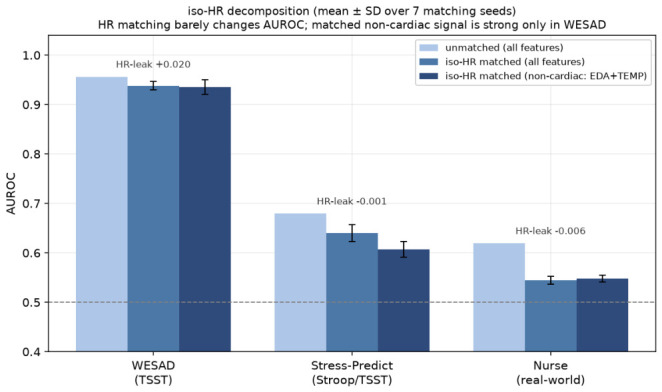
Iso-HR decomposition (bars: unmatched all-feature, iso-HR matched all-feature, and iso-HR matched non-cardiac [EDA+temperature]; error bars are standard deviations (SDs) over seven matching seeds). Heart-rate matching barely changes AUROC (HR-leakage ≈ 0), and the matched non-cardiac signal is strong only for laboratory social-evaluative stress (WESAD). The two weak-signal corpora remain floor-effect-limited and do not support causal attribution.

The iso-HR decomposition (Table 10, Figure 7) gives an HR-leakage index of ≈0 in every corpus (WESAD +0.020±0.008, Stress-Predict −0.001±0.006, Nurse −0.006±0.006 over seven matching seeds): HR level adds essentially nothing once the other signals are present. As cautioned in the Methods, a near-zero index is informative only where a signal exists. The decisive case is WESAD, where matching HR barely changes performance (all-feature AUROC 0.955→0.938±0.009), and the purely non-cardiac (EDA+temperature) signal remains strong after matching (0.935±0.015); this argues against an HR confound as the explanation for WESAD’s signal. For Stress-Predict and Nurse, the matched non-cardiac AUROC is near chance (0.607±0.016 and 0.548±0.007), so for the near-zero leakage, there is a floor effect: nothing discriminates, including HR. Taken together, the WESAD result argues against HR-level confounding as the main explanation for its signal. The weak Stress-Predict and Nurse signals do not distinguish among label noise, device fit, stressor type, population, and workflow context. The small standard deviations across seeds confirm that this bounded decomposition is not a single-draw artifact.

### 3.7. The Nurse Evaluation Shows Weak Operational Discrimination

**Figure 8:**
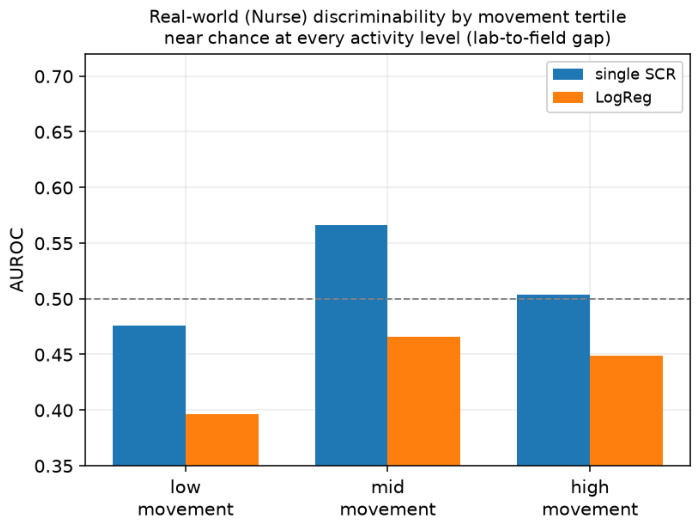
Real-world (Nurse) discriminability by movement tertile. AUROC remains near chance at all activity levels; movement stratification does not resolve the source of weak discrimination. The low/middle/high tertiles contain 3076/3075/3075 windows, respectively, and all 15 Nurse subjects are represented in each tertile.

Stratifying Nurse windows into movement tertiles leaves AUROC near chance throughout (Figure 8: single-feature 0.476/0.566/0.504 for low/mid/high movement; logistic below 0.5). The absence of a monotonic movement effect means motion alone does not account for the pattern, but it does not identify whether weak physiology, device fit, context, or retrospective labels dominate.

**Figure 9:**
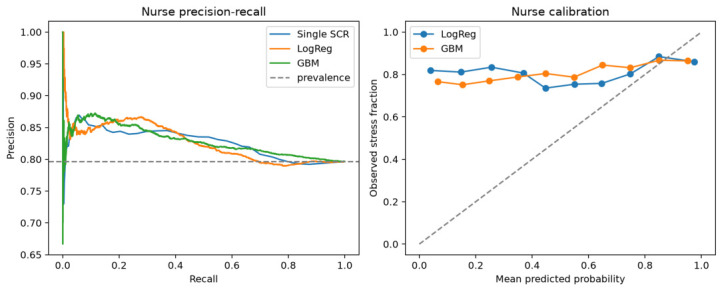
Nurse precision–recall curves (**left**) and reliability curves for the probabilistic source-trained models (**right**). The dashed precision baseline is the target prevalence (0.796); the diagonal is perfect calibration.

The operational metrics reinforce this uncertainty (Table 11, Figure 9). The average precision of 0.826–0.828 is only 0.030–0.032 above the stress prevalence of 0.796. At the fixed 0.5 threshold, logistic regression detects 58.3% of stress windows but falsely flags 53.5% of non-stress windows; gradient boosting lowers the false-positive rate to 27.5% while detecting only 35.6% of stress windows. Calibration is also poor (ECE 0.238 and 0.378). These values do not support an operational alerting system under the present labels. Because the labels are sparse, retrospective, and imbalanced, they also do not establish that the underlying physiology cannot transfer.

### 3.8. Error Stratification Locates the Residual Nurse Discrimination

**Figure 10:**
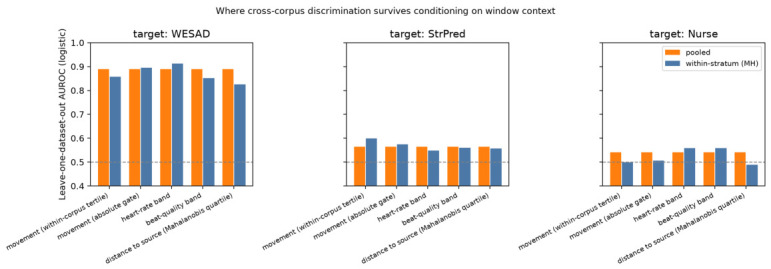
Pooled versus within-stratum leave-one-dataset-out AUROC for the logistic model. For Nurse, conditioning on movement level or on distance to the source distribution removes the residual discrimination entirely. MH denotes Mahalanobis-distance stratification in the figure.

To describe where transfer errors concentrate, we stratified the fixed source-trained scores—without refitting—by movement level, HR band, beat quality, and Mahalanobis distance to the source feature distribution, and combined the within-stratum AUROCs with $n_{\mathrm{stress}}\times n_{\mathrm{non-stress}}$ weights (Table 12, Figure 10). The quantity of interest is the gap between the pooled and the within-stratum estimate: the part of apparent discrimination that comes from score offsets between strata rather than ranking inside them.

For Nurse, that gap consumes the entire effect. Pooled logistic AUROC is 0.541, but conditioning on distance to the source distribution leaves 0.489, and conditioning on movement tertile leaves 0.499—at or below chance in both cases. The already weak Nurse signal is therefore largely a between-context offset rather than within-context ranking. The per-subject picture agrees: of 15 Nurse subjects, 13 have both classes, and their logistic AUROC has median 0.511 and range 0.370–0.725, so roughly half fall below chance.

WESAD behaves differently: conditioning reduces AUROC from 0.890 to 0.826 (distance) and 0.857 (movement) but leaves it clearly above chance, and 15 of 15 subjects are evaluable with median 0.985. One WESAD stratum is a useful caution, however: using the externally derived stress-window 95th-percentile gate defined in Section 2.6, the 102 windows at or above 0.0796 g show that discrimination collapses to 0.530 (single) and 0.467 (logistic), so even the strongest corpus loses its signal once participants are physically active.

Stress-Predict shows no comparable concentration; every stratified estimate stays within 0.036 of the pooled value.

These strata are observed conditions, not manipulated factors, so a difference between strata is an association and does not by itself identify a cause.

## 4. Discussion

This study reframes wearable stress detection as a clinical-informatics translation problem. The key result is not simply that one model outperformed another. Rather, the same physiological measurements that appear useful in a controlled stress protocol become weak and unstable when moved into a physically active nursing workflow. For Healthcare applications, this distinction is critical: a stress score deployed in a ward would not be consumed as an abstract AUROC value, but as a possible alert, dashboard signal, or workforce-management input. If such a signal is not transferable, it can add interruption and operational noise instead of helping clinicians. In the language of digital medicine validation, the present findings are an analytical and translational warning: a wearable-derived measure may be technically computable and within-dataset predictive, yet still not be fit for a new clinical context of use [8,9].

Four findings define the boundary. First, added model complexity did not solve the translation problem. A single SCR feature matched or exceeded logistic regression, histogram boosting and four unsupervised adaptation methods where the signal was strong, and none of them produced reliable Nurse discrimination. The one exception is instructive rather than encouraging: best-case subspace alignment beat the single feature on Nurse by 0.020 AUROC, but only after its component count was chosen on target performance, and its subject-bootstrap interval still spans chance. Adversarial representation learning remains untested, and supplying target labels directly did not stabilize Nurse either; what the present evidence shows is that complexity alone is not evidence of transfer when the target labels and signal are weak.

This finding aligns with the broader literature on wearable stress monitoring, which increasingly recognizes that model generalization, labeling quality, and context are central limitations rather than minor implementation details [22,23]. Studies in office-like environments and cross-context prediction frameworks show that behavioral and contextual signals may carry information that physiological channels alone do not capture [24,26]. Our results extend that logic to nursing work: the absence of reliable Nurse-corpus discrimination should not be read as a failure of EDA as a signal in general. Under sparse retrospective labels it is equally compatible with weak physiology, label mismatch, device fit, and an under-specified workflow model.

Second, iso-HR matching shows that the result cannot be reduced to a simple heart-rate-confounding story. In WESAD, where a strong signal exists, the non-cardiac EDA+temperature signal remains strong after HR matching. This supports the physiological plausibility of EDA-based stress detection under a social-evaluative laboratory stressor. In Stress-Predict and Nurse, however, all matched feature sets remain weak. The near-zero HR-leakage index in these corpora is therefore a floor effect rather than proof of robust stress specificity. The interpretation is clinically important: the problem is not only that HR shortcuts can mislead models but also that the two weak corpora do not provide enough discrimination to identify a single explanation for transfer failure.

This point also sharpens the role of physical activity. Recent acute stress-versus-exercise data demonstrate that stress and exercise can be measured with overlapping wrist channels while still requiring explicit contextual separation [27]. Our iso-HR result is consistent with that view. HR matching is necessary but not sufficient: it removes the most direct cardiac shortcut, yet it cannot fully resolve ambiguity introduced by movement, task demands, thermal context, and retrospective labeling in a real clinical shift.

Third, the Nurse corpus should be treated as an ecologically demanding context-of-use test rather than as a failed benchmark or a nurse-specific prediction target. Its physiological recordings are embedded in patient care, movement, interruptions, and delayed self-report. In the original Nurse dataset, candidate stress events were first flagged by a detection algorithm and then validated retrospectively by nurses in an end-of-shift survey; the survey also allowed additional events to be reported, but none were added [29]. Minute-by-minute physiological triggers are therefore not independently and continuously annotated. A label describing high stress across a multi-hour period may mix acute psychological strain, physical workload, baseline fatigue, and contextual pressure. Under this labeling structure, a wearable model may be asked to solve a problem that the available ground truth cannot cleanly define. This does not make the Nurse result proof of physiological non-transfer; it makes the corpus a realistic test of both measurement and ground-truth limitations in field-based clinical informatics.

This interpretation follows from the operational definition in Table 3. The three corpora are deliberately used as different context-of-use probes rather than as exchangeable sources of more training data. WESAD asks whether a clear physiological stress signal is detectable under controlled conditions; Stress-Predict asks whether that signal survives a shift to a Stroop task and an attenuated TSST implementation; Nurse asks whether the same class of wearable output remains meaningful when embedded in clinical work. Under this definition, low Nurse-corpus transfer is not a secondary inconvenience. It is a healthcare validity warning, but the wide subject interval and label limitations prevent a definitive physiological conclusion. A model that works in wellness-like conditions but remains weak in this boundary case should not be interpreted as ready for healthcare workforce dashboards or alerts.

Fourth, the bounded robustness analyses do not reveal a simple technical repair. Non-overlapping and 300 s windows preserve the transfer pattern, six HRV/quality features change AUROC by at most 0.017, plausible Stress-Predict label shifts do not create strong discrimination, and four unsupervised adaptation methods leave the weak corpora weak. Error stratification adds the one positive localization we can make: for Nurse, conditioning on movement level or on distance to the source distribution removes the residual discrimination entirely (Section 3.8), so what little the pooled estimate contains is a between-context offset rather than within-context ranking. The marginal feature shifts, and the strata remain descriptive rather than causal, but together they point away from a missing-model explanation and toward context and label quality.

### 4.1. Interpreting Negative Transfer as Healthcare Evidence

Negative transfer is often treated as an engineering problem: the model did not generalize, so the next step is to add more features, more data, or a stronger domain-adaptation method. We took two of those steps. Three additional unsupervised adaptation methods (Section 3.3) and an accelerometer context-aware model (Section 3.4) left the weak corpora weak, and the context features actively reduced transfer because they encode protocol rather than physiology. That interpretation is therefore partly correct, but incomplete for healthcare. In a context-of-use framework, failure to transfer is itself a form of evidence. It identifies the boundary at which a digital measure no longer represents the construct that users may believe it represents [8,9]. For a consumer wellness score, this boundary may be acceptable if the output is used only for personal reflection. For a healthcare workforce signal, the same boundary has practical meaning because the output may be interpreted as a marker of work strain, risk, or need for intervention.

This distinction is important for the present findings. The Nurse corpus does not merely lower the average cross-corpus AUROC. It changes the interpretation of the entire pipeline. A model that performs well on WESAD but fails under naturalistic nursing work is not simply a model with poor external validation; it is a model whose apparent construct validity is conditional on a narrow stressor and activity context. The high WESAD result shows that wrist EDA can carry a detectable stress signal under a strong, well-defined social-evaluative protocol. The weak Nurse result shows that this signal does not automatically remain a healthcare-workflow measure. Both results are necessary for the translational claim. Without the positive benchmark result, there would be little physiological signal to translate. Without the negative healthcare boundary result, the benchmark could be overread as deployment evidence.

This also affects how future model improvements should be judged. A more complex classifier, additional sensor channels, or domain adaptation may improve cross-corpus performance, but such improvement would not by itself establish clinical utility [45,46]. The improved model would still need to show what construct it measures in the clinical workflow, whether its errors cluster around predictable tasks, and whether its output changes a decision in a beneficial way. Conversely, a simple model may be preferable if it has a narrower but more transparent use, for example, identifying intervals of physiological arousal for later voluntary reflection rather than generating real-time alerts. The methodological lesson is therefore not that public nurse datasets are too noisy to be useful. It is that their noise, sparsity, and workflow complexity are precisely what reveal the gap between benchmark validity and healthcare validity.

These findings connect directly to patient-safety and human-factors concerns. Healthcare already struggles with alert burden, repeated alerts, cognitive load, and alarm fatigue [15,16,47,48]. A consumer smartwatch stress score or wellness dashboard signal that mistakes physical work for psychological strain could become another low-specificity notification stream. This concern is not hypothetical in healthcare workforce contexts, where workload, burnout, staffing, and alarm burden are already linked to staff well-being, perceived safety, care quality, and patient safety [12,13,15,16]. Even if such a system is framed as staff wellness rather than patient monitoring, its outputs could influence attention, self-perception, staffing dashboards, or occupational health decisions. The appropriate standard is therefore higher than within-dataset classification accuracy. A wearable stress metric intended for healthcare workflows should demonstrate transfer across stressor types, robustness under HR control, and acceptable false-positive behavior at realistic field prevalence.

### 4.2. Clinical and Informatics Implications

The clinical utility question is therefore narrower, and more demanding, than the usual benchmark question. A wrist-derived stress score is not a diagnosis, a validated workload measure, or an immediately actionable alert by itself. Its value depends on whether the score improves a specified healthcare decision without adding cognitive, operational, or interpretive burden. In a private wellness setting, a noisy stress estimate may be tolerable because the user can ignore it. In a healthcare workforce setting, the same signal may be routed into dashboards, staffing discussions, occupational-health interpretations, or well-being interventions. The relevant evidence standard is therefore not only “Can the model distinguish labeled stress windows?” but “Does the measure remain meaningful, specific, and useful in the intended clinical workflow?” [8,9,45].

The single-SCR result has a direct design implication. If a fixed, physiologically interpretable feature performs on par with logistic regression or gradient boosting where a stress signal exists, then added model complexity requires its own justification. For healthcare workforce applications, a simpler feature can reduce computation, ease on-device or edge deployment, and make the output easier to audit [24]. It also gives clinicians, occupational-health teams, and system designers a clearer account of what the device is reacting to: discrete skin-conductance responses rather than an opaque weighted combination of many features. This does not mean that multivariate models are unnecessary in all future systems, but it does mean that complexity should be added only when it improves transfer, calibration, false-alert behavior, or decision value beyond an interpretable physiological baseline.

This is a shift from benchmark validity to healthcare context-of-use validity, as set out by digital-measure validation frameworks [8,9] and established prediction-model evaluation methods [45,46], and summarized against the corresponding validation requirements in Table 13. The criteria also clarify why the Nurse corpus is informative even when AUROC is low. Poor transfer in this setting indicates that the measure cannot yet support clinical workforce interpretation without additional context, not simply that one dataset is difficult.

### 4.3. Deployment Failure Modes and Prospective Validation Needs

The practical implication is not that wrist wearables have no role in healthcare workforce monitoring. Instead, they should be embedded in context-aware informatics designs that explicitly anticipate deployment failure modes [20]. The first failure mode is a false-positive workflow signal. A wrist model may flag patient transfer, rapid walking to a call light, donning or doffing equipment, or heat exposure as stress. In a private wellness application, such a signal may be annoying but inconsequential. In a clinical workforce setting, repeated false-positive scores could become another low-specificity notification stream, contribute to alert burden, or create avoidable concern about normal clinical activity [15,16,47,48]. The second failure mode is a false-negative emotional signal. A clinician may experience distress during a difficult conversation, conflict, error recovery, or end-of-life care without producing the same physiological pattern as a laboratory stressor. A model that misses such events could provide misleading reassurance if the score is interpreted as a workload or well-being indicator.

A third failure mode is aggregation error. Stress scores are often easier to display at the shift, unit, or staff-group level than to interpret at the minute-by-minute level. However, aggregating noisy physiological estimates can make them appear more stable and objective than the underlying labels justify. This is especially important for healthcare workforce monitoring because the same dashboard value could be read as a personal wellness trend, an occupational health concern, a staffing signal, or a proxy for care pressure. The fourth failure mode is governance drift: a signal introduced for individual reflection may gradually become part of performance management, staffing review, or organizational surveillance. Even when no patient data are involved, workforce stress monitoring is not ethically neutral because it measures clinicians during work and may be interpreted by people other than the wearer [49,50]. These risks make the “intended use” of the measure a methodological variable, not only a deployment detail.

Future systems may therefore need to combine physiology with passive context such as activity state, shift timing, task category, location within the care environment, or workload metadata. Rather than one monolithic stress model, clinical systems may require modular submodels that interpret physiological deviations within the operational context in which they occur. Such designs would better align with the realities of nursing work, where the same HR or EDA pattern may mean patient transfer, urgent response, heat exposure, anxiety, or emotional distress depending on context. Table 13 translates this argument into validation requirements for future Healthcare-oriented wearable stress studies, alongside the benchmark framing each requirement replaces.

These requirements also imply a different reporting standard from conventional classification papers. The AUROC remains useful for separating models, but it is insufficient for deciding whether a stress score should appear in a clinical workflow. A healthcare-facing evaluation should report calibration [46] and decision-analytic net benefit [45], and, as we argue here, expected false-positive counts per shift, positive predictive value under field prevalence, and performance within clinically relevant activity states. A model with an acceptable AUROC can still be unusable if it fires repeatedly during normal rounding, patient transfers, or alarm response. Conversely, a modest classifier may be useful if it is restricted to a narrow action pathway, such as prompting optional self-reflection after a high-burden interval, and if it remains silent during predictable physical work.

Threshold selection is therefore part of clinical design rather than a purely statistical afterthought. In wellness applications, a threshold can be tuned for user engagement or subjective plausibility. In healthcare workforce settings, the same threshold defines who is interrupted, what is shown to supervisors or occupational-health teams, and how often normal care activity is reframed as stress. Future studies should therefore prespecify whether outputs are individual-only, aggregated for unit-level monitoring, or connected to an action by another party. Without that specification, model performance cannot be translated into clinical utility because the cost of an error is undefined [45]. The separate ethical requirements of privacy, transparency, consent, and accountability remain essential when workforce signals are collected or shared [49,50].

This framing also changes what a successful next study should look like. Rather than optimizing a single model on one corpus, future Healthcare-oriented wearable stress studies should prespecify the intended decision context, collect time-resolved labels close to clinically meaningful events, quantify false-alert costs, and evaluate whether physiological signals add value over contextual baselines. Such a design would move the field from “Can a model classify stress in a benchmark?” toward “Can this digital measure support a specific clinical or workforce decision without increasing burden?”

### 4.4. Limitations

Several limitations bound these conclusions. First, the strongest positive evidence rests on WESAD, which has only 15 subjects; although subject-bootstrap intervals exclude chance, small-sample inference warrants caution and the precise AUROC values should be read with their intervals, not as three-decimal point estimates. Second, the Nurse labels are retrospective, self-reported, sparse and imbalanced (∼80% stress), and real movement is present. The near-chance result therefore reflects label noise, construct heterogeneity, and weak signal separability; it is not a pure physiological statement about EDA, HR, or temperature. Third, iso-HR matching is a random subsample. We mitigate single-draw variance by repeating over seeds, but matching also reduces sample size, especially for WESAD (n=318 matched windows), and it cannot remove all activity, posture, or task-context differences.

Fourth, the binary stress/non-stress reduction and differences in protocol, device fit, population, labeling cadence, and work setting across corpora limit direct comparison. These differences are partly the object of study, because cross-corpus heterogeneity is central to context-of-use validity, but they also prevent a clean causal attribution of transfer failure to any single factor. Fifth, the four adaptation methods evaluated here are all shallow and transductive. Adversarial representation learning [44,51] was not evaluated, and with 15, 35 and 15 subjects per corpus, a deep adaptation experiment would not be adequately powered; the supervised reference we do report supplies target labels as an upper-bound check rather than implementing a dedicated few-shot method; the present result must therefore not be generalized to all adaptation methods. Likewise, the context-aware model uses the only context channel the wearable provides. Shift metadata, patient acuity, alarm exposure and workload were unavailable in these public corpora and could behave differently. Sixth, the 300 s sensitivity improves the interpretability of HRV but sharply reduces the number of windows; it is a robustness analysis rather than a replacement for the 64 s primary protocol. Seventh, session-level EDA decomposition introduces a within-subject look-ahead; this is not a cross-subject leak and does not inflate LOSO estimates, but it does not guarantee identical online performance in a prospective system.

Eighth, moving Stress-Predict protocol intervals by up to 64 s bounds only one form of alignment error. Ninth, marginal Wasserstein distances identify feature distribution discrepancies but cannot isolate stressor, population, device fit, activity, or labeling as causal drivers.

Finally, this study is a secondary analysis of public data, not a prospective healthcare deployment trial. It does not evaluate user-facing dashboards, alert thresholds, acceptability, privacy governance, clinician response, staffing decisions, or occupational-health workflows. It also does not test whether a wearable stress output improves decisions beyond contextual baselines already available in hospitals, such as shift timing, task load, alarm exposure, patient acuity, or staffing ratios. The conclusions should therefore be read as boundary-setting evidence for translational validation, not as a final clinical evaluation of a specific stress-monitoring product. The appropriate next step is not merely a larger benchmark but a prospective context-of-use study that combines time-resolved labels, activity and workload annotation, false-alert analysis, and a prespecified action pathway. Such a study could reduce the current Nurse-label mismatch by collecting event-proximal or continuous visual analog scale (VAS) stress ratings and linking them, under appropriate governance, to electronic health record (EHR)-derived workload, acuity, alarm, and task metadata.

## 5. Conclusions

Across three public wrist-E4 corpora, a single SCR feature was on par with the multivariate models where a stress signal existed, while cross-corpus transfer remained weak for Stress-Predict and Nurse. Four unsupervised adaptation methods did not change that, and adding wrist-accelerometer activity context raised within-corpus performance while lowering every transfer estimate, because the movement–stress association is protocol-specific. Non-overlap, longer-window, HRV-ablation, and label-alignment checks preserved the same pattern. Iso-HR evidence was diagnostic mainly in WESAD, where a strong matched non-cardiac signal argues against HR-level confounding; floor effects prevent the same inference in the two weak-signal corpora. The present Nurse labels yield weak, uncertain operating performance but cannot distinguish physiological non-transfer from sparse, retrospective, imbalanced ground truth.

Wearable stress analytics may still become useful in healthcare, but only if they are validated as workflow-sensitive informatics tools rather than standalone consumer metrics. Before such systems are used to generate alerts, dashboards, or occupational-health interpretations for nurses and other clinicians, they should be evaluated under HR control, across stressor types, and within the physical and cognitive context of clinical work.

## Figures and Tables

**Table 1:** Analysis pipeline and design rationale.

Step	Purpose and Implementation
Data sources	Use three public Empatica E4 corpora spanning laboratory social-evaluative stress (WESAD), a Stroop and attenuated Trier Social Stress Test (TSST) interview protocol (Stress-Predict), and real-world hospital nurse work (Nurse).
Windowing	Segment wrist signals into 64 s windows with 50% overlap to obtain comparable physiological epochs across corpora.
Signal processing	Extract HR/HRV, EDA, temperature, and accelerometer-derived quantities; decompose EDA into tonic and phasic components and count SCR events.
Feature design	Compare one fixed, interpretable SCR feature with logistic regression and histogram gradient boosting; evaluate CORAL, subspace alignment, transfer component analysis, importance weighting, a supervised target-label reference, and an accelerometer context-aware variant.
Transfer control	Use subject-disjoint validation within corpora and leave-one-dataset-out testing across corpora to distinguish benchmark performance from translation.
HR-confound control	Apply iso-HR matching within subject, then compare all-feature, HR-level-free, and non-cardiac feature sets using the HR-leakage index.
Uncertainty	Report subject-cluster bootstrap intervals, paired subject-bootstrap model differences, and cluster-aware permutation tests.
Healthcare interpretation	Interpret weak Nurse-corpus transfer as a workflow-validity warning while separating physiological non-transfer from sparse, retrospective ground-truth limitations.

**Table 2:** Cross-corpus evaluation datasets (wrist Empatica E4).

Corpus	Windows	+/−	Subjects	Stressor
WESAD	1133	287/846	15	TSST (social-evaluative)
Stress-Predict	2846	952/1894	35	Stroop + attenuated TSST interview
Nurse	9226	7347 /1879	15	Real-world hospital (self-report)

**Table 3:** Operational role of each corpus in the context-of-use analysis.

Corpus	Operational Context	Validity Question	Interpretation of Failure
WESAD	Controlled social-evaluative laboratory stress.	Can wrist physiology classify a strong, protocol-defined stressor under benchmark conditions?	Weak performance would challenge the basic physiological signal.
Stress-Predict	Stroop and an attenuated TSST interview in a structured protocol.	Does the signal survive a shift in stressor type and labeling protocol?	Weak performance suggests stressor-dependent physiology or label-alignment limits.
Nurse	Naturalistic hospital work with movement, interruptions, alarms, shift demands, and retrospective self-report.	Does a wellness-like wearable stress measure retain meaning in a healthcare workforce workflow?	Weak performance indicates unsupported healthcare translation without richer context, not a nurse-specific model failure.

**Table 4:** Within-corpus leave-one-subject-out AUROC. A single skin-conductance feature is on par with machine learning where the signal is strong (WESAD); machine learning shows only a modest edge on Stress-Predict, where all models remain weak. Values are LOSO point estimates; uncertainty for the transfer claims is given in Table 5.

Within-Corpus (LOSO)	Single (SCR)	Logistic	Gradient Boosting
WESAD	0.897	0.876	0.886
Stress-Predict	0.563	0.628	0.633
Nurse	0.553	0.450	0.489

**Table 6:** Leave -one-dataset-out AUROC by unsupervised adaptation method, with 95% subject-bootstrap confidence intervals. Subspace alignment and transfer components use the target-maximizing hyperparameters (components, and for transfer components also the kernel bandwidth), an optimistic upper bound. The final column is the paired subject-bootstrap difference (single SCR minus method); an interval containing zero means the tested comparison does not separate them. Paired differences are subject-cluster bootstrap means and need not equal the difference of the two rounded point estimates.

Target	Method	AUROC [95% CI]	Single − Method [95% CI]
WESAD	Single SCR (reference)	0.897 [0.832–0.953]	—
	Logistic, no adaptation	0.890 [0.814–0.949]	+0.007 [−0.003, +0.022]
	CORAL	0.879 [0.829–0.930]	+0.018 [−0.007, +0.036]
	Subspace alignment	0.895 [0.818–0.954]	+0.003 [−0.005, +0.017]
	Transfer components	0.865 [0.810–0.921]	+0.030 [−0.000, +0.065]
	Importance weighting	0.897 [0.850–0.946]	−0.000 [−0.022, +0.014]
Stress-	Single SCR (reference)	0.563 [0.525–0.607]	—
Predict	Logistic, no adaptation	0.564 [0.530–0.602]	−0.001 [−0.015, +0.015]
	CORAL	0.564 [0.531–0.602]	−0.001 [−0.016, +0.014]
	Subspace alignment	0.567 [0.531–0.609]	−0.004 [−0.014, +0.007]
	Transfer components	0.565 [0.529–0.602]	−0.002 [−0.024, +0.021]
	Importance weighting	0.565 [0.532–0.603]	−0.002 [−0.020, +0.017]
Nurse	Single SCR (reference)	0.553 [0.407–0.694]	—
	Logistic, no adaptation	0.541 [0.423–0.656]	+0.009 [−0.037, +0.049]
	CORAL	0.542 [0.417–0.663]	+0.009 [−0.016, +0.034]
	Subspace alignment	0.574 [0.431–0.707]	−0.020 [−0.039, −0.002]
	Transfer components	0.571 [0.421–0.701]	−0.017 [−0.063, +0.026]
	Importance weighting	0.543 [0.423–0.658]	+0.007 [−0.035, +0.044]

**Table 7:** Wrist -accelerometer context-aware model. Entries are AUROC without/with the four activity features, all else held fixed. The final column is the signed univariate AUROC of mean absolute jerk against the stress label: above 0.5 means more movement in stress windows, below 0.5 means less.

	Within-Corpus (LOSO)		Transfer (LODO)	Movement–Label
Corpus	Logistic	Boosting	Logistic	AUROC (Jerk)
WESAD	0.876/0.898	0.886/0.930	0.890/0.836	0.828
Stress-Predict	0.628/0.681	0.633/0.671	0.564/0.520	0.481
Nurse	0.450/0.468	0.489/0.572	0.541/0.519	0.590

**Table 8:** Windowing sensitivity of leave-one-dataset-out AUROC. Each entry is single SCR/logistic regression; all other settings are fixed.

Window/Step	WESAD	Stress-Predict	Nurse
64/32 s (primary)	0.897/0.890	0.563/0.564	0.553/0.541
64/64 s (non-overlap)	0.896/0.890	0.564/0.563	0.554/0.545
300/150 s	0.917/0.913	0.562/0.556	0.564/0.522

**Table 9:** HRV-feature sensitivity for within-corpus LOSO histogram gradient boosting. Entries are all features/the same features excluding six HRV and quality variables. Differences quoted in the text are computed before rounding, so they need not equal the difference of the two rounded entries.

Corpus	64 s	300 s
WESAD	0.955/0.938	0.959/0.965
Stress-Predict	0.675/0.672	0.661/0.651
Nurse	0.621/0.616	0.637/0.648

**Table 10:** Per-corpus iso-HR decomposition (AUROC). “Unmatched (all)” is deterministic; matched columns are mean ± standard deviation (SD) over seven matching seeds (the matched subsample is random). “Non-cardiac” is EDA + temperature only. The HR-leakage index = matched (all) − matched (HR-level-free), where “HR-level-free” excludes mean HR and mean IBI but still retains cardiac dynamics and HRV; it is near zero everywhere, while the matched non-cardiac signal is strong only for social-evaluative stress.

Corpus	Unmatched	Matched	Matched	HR-Leakage
	(All)	(All)	(Non-Cardiac)	Index
WESAD	0.955	0.938±0.009	0.935±0.015	+0.020±0.008
Stress-Predict	0.675	0.640±0.017	0.607±0.016	−0.001±0.006
Nurse	0.621	0.544±0.008	0.548±0.007	−0.006±0.006

Matched windows *n*: WESAD 318, Stress-Predict 1524, Nurse 2210.

**Table 11:** Nurse leave-one-dataset-out operational metrics. Logistic regression and gradient boosting are trained on WESAD and Stress-Predict; threshold 0.5 is fixed without Nurse-label tuning. ECE is 10-bin expected calibration error.

Model	AUROC	AP	Brier	ECE	Bal. Acc.	Sens.	FPR
Single SCR	0.553	0.826	–	–	–	–	–
Logistic	0.541	0.826	0.243	0.238	0.524	0.583	0.535
Boosting	0.559	0.828	0.344	0.378	0.541	0.356	0.275

**Table 12:** Leave -one-dataset-out error stratification for the logistic model. “Within-stratum” combines stratum-specific AUROCs with $n_{\mathrm{stress}}\times n_{\mathrm{non-stress}}$ weights; “between-stratum share” is pooled minus within-stratum. A large positive share means the pooled value is carried by score offsets between strata rather than by ranking inside them. The 0.0796 g movement gate is the externally derived stress-window 95th percentile defined in Section 2.6.

Target	Stratifier	Pooled	Within-Stratum	Between-Stratum Share
WESAD	Movement tertile	0.890	0.857	+0.033
	Movement gate (0.0796 g)	0.890	0.895	−0.005
	HR band	0.890	0.912	−0.023
	Beat quality	0.890	0.851	+0.038
	Distance to source	0.890	0.826	+0.064
Stress-Predict	Movement tertile	0.564	0.600	−0.036
	Movement gate	0.564	0.574	−0.011
	HR band	0.564	0.549	+0.015
	Beat quality	0.564	0.561	+0.003
	Distance to source	0.564	0.557	+0.007
Nurse	Movement tertile	0.541	0.499	+0.042
	Movement gate	0.541	0.506	+0.036
	HR band	0.541	0.559	−0.018
	Beat quality	0.541	0.559	−0.018
	Distance to source	0.541	0.489	+0.052

**Table 13:** From benchmark validity to healthcare context-of-use validity: the requirement, rationale, and example design for each validation dimension before healthcare workforce deployment.

Validation Dimension	Benchmark Framing	Healthcare Context-of-Use Requirement	Healthcare Rationale	Example Study Design
Construct validity	Classify stress labels within a known corpus.	Determine whether the wearable signal represents a clinically interpretable state under physical work, interruptions, alarms, and delayed self-report; prespecify whether the target is acute stress, workload, fatigue, distress, or a broader well-being state.	Different constructs imply different actions and different risks of misinterpretation.	Combine time-stamped self-report with event-level clinical annotations.
Activity robustness and transfer	Use subject-disjoint cross-validation within one dataset.	Demonstrate transfer across stressor types, HR distributions, activity contexts, and field labeling conditions, and show that performance survives HR control, movement context, posture, and shift-related physical work.	Physical care tasks can resemble stress physiology at the wrist.	Evaluate models within matched activity strata and report false positives during patient-care tasks.
Workflow specificity	Report a model that performs well on public data.	Prespecify the intended use, users, action pathway, and false-alert tolerance: where the score appears, who sees it, and what action it supports.	A score used for private reflection has a different burden than a staffing or occupational-health signal.	Prospectively test a dashboard or alert pathway with prespecified users and actions.
Incremental decision value	Maximize discrimination metrics such as AUROC.	Show that the measure supports a specified workflow, occupational-health, or well-being decision beyond contextual baselines such as shift type, workload, task category, or alarm exposure.	A wearable measure should add decision value beyond information already available in the work system.	Report whether wearable features improve calibration, discrimination, or decision curves over contextual models.
False-alert burden	Misclassification lowers model performance.	Quantify alert frequency, false-positive clusters, and user response under field prevalence, recognizing that ambiguous scores add alert burden, staff concern, and dashboard noise.	Even accurate models can fail if they create interruption or dashboard noise.	Use prospective monitoring with alert suppression rules and workload sensitive thresholds.
Governance and privacy	Not addressed in benchmark reporting.	Specify data access, retention, aggregation, consent, and non-punitive use.	Workforce monitoring can affect trust even when framed as wellness support.	Evaluate acceptability with clinicians, managers, and occupational health stakeholders before deployment.

## Data Availability

All datasets are publicly available. WESAD is available through the University of California, Irvine (UCI) Machine Learning Repository and is described in Schmidt et al. [3,52]. Stress-Predict is available through the authors’ public repository and its dataset article [28]. The Nurse dataset is available through Dryad and the Scientific Data descriptor [29,53]. Analysis code, the exact environment, and reproducible shell commands are openly available at https://github.com/RURUGURU/isohr-wearable-stress (accessed on 3 August 2026) under the MIT licence and are archived at Zenodo, https://doi.org/10.5281/zenodo.21638616 (accessed on 3 August 2026). That concept DOI always resolves to the most recent release; the version evaluated here is v1.0.0, https://doi.org/10.5281/zenodo.21638617 (accessed on 3 August 2026). The PhysioNet dataset used only to derive the fixed external movement threshold is publicly available as described by Hongn et al. [43].

## Abbreviations

The following abbreviations are used in this manuscript:

AUROC	area under the receiver operating characteristic curve
CI	confidence interval
AP	average precision
CORAL	correlation alignment
EDA	electrodermal activity
ECE	expected calibration error
EHR	electronic health record
EMA	ecological momentary assessment
FPR	false-positive rate
HR	heart rate
HRV	heart-rate variability
IBI	inter-beat interval
iso-HR	iso-heart-rate
LODO	leave-one-dataset-out
LOSO	leave-one-subject-out
pNN50	percentage of successive heartbeat intervals differing by >50 ms
RMSSD	root mean square of successive differences
SD	standard deviation
SDNN	standard deviation of normal-to-normal intervals
SCL	skin conductance level
SCR	skin conductance response
TCA	transfer component analysis
TSST	Trier Social Stress Test
VAS	visual analog scale
WESAD	Wearable Stress and Affect Detection
